# Supplementary material for: Genetic Diversity and Connectivity of Ocypode ceratophthalmus in the East and South China Seas and Its Implications for Conservation
Source: Biology (Basel). 2023 Mar 12;12(3):437. doi: 10.3390/biology12030437 (PMC10044906; doi:10.3390/biology12030437)
Supplement: Supplementary file 1 [file biology-12-00437-s001.zip › biology-2195983-supplementary.pdf]

Supplementary Information

Genetic Diversity and Connectivity of *Ocypode ceratophthalmus* in the East and South China Seas and Its implications for conservation

Feng Zhao, Yue Liu , Zihan Wang, Jiaying Lu, Ling Cao, and Cong Zeng *

School of Oceanography, Shanghai Jiao Tong University, Shanghai 200030, China
* Correspondence: congzeng@sjtu.edu.cn

Table S1. Current status of genetic connectivity between populations in the East China Sea and populations in the South China Sea. The Web of science and CNKI were used to search the published papers. ((genetic OR gene) AND (" East China Sea" AND "South China Sea ")) were used as search keywords for English articles, and ("East China Sea", "South China Sea" and "genetic") (in Chinese) were used as search keywords for Chinese articles. The search deadline was December 29, 2022. Only articles in which the studies species had sampling sites in the East China Sea and the South China Sea were retained. A case was considered to have significant genetic differentiation if there was at least one significant value of the fixation index (F_{ST}) between populations in the East China Sea and populations in the South China Sea.

ID	Species	Genetic marker	Genetic differ-enti-ation	Number of popula-tions	Reference
1	*Pampus chinensis*	*COI*	Yes	6	Population genetic structure and genetic diversity of Chinese pomfret at the coast of the East China Sea and the South China Sea
2	*Pagrus major*	*SSR*	Yes	2	Genetic Structure of Red Sea Bream (*Pagrus majo*r) Population off Japan and the Southwest Pacific, Using Microsatellite DNA Markers
3	*Eleutheronema tetradactylum*	*Cytb*	Yes	4	Low mtDNA *Cytb* diversity and shallow population structure of *Eleutheronema tetradactylum* in the East China Sea and the South China Sea
4	*Thamnaconus hypargyreus*	*D-Loop*	No	5	Shallow mitochondrial phylogeographical pattern and high levels of genetic connectivity of *Thamnaconus hypargyreus* in the South China Sea and the East China Sea
5	*Eleutheronema rhadinum*	*COI*	No	2	Genetic diversity and population structure of *Eleutheronema rhadinum* in the East and South China Seas revealed in mitochondrial *COI* sequences
6	*Nibea albiflora*	*COI*	No	3	Genetic diversity and population structure of *Nibea albiflora* in the

					China Sea revealed by mitochondrial *COI* sequences
7	*Epinephelus akaara*	*D-Loop*	Yes	6	Genetic population structuring and demographic history of red spotted grouper (*Epinephelus akaara*) in South and East China Sea
8	*Scomber japonicus*	*SSR*	Yes	6	Microsatellite analysis reveals the population structure and migration patterns of *Scomber japonicus* (Scombridae) with continuous distribution in the East and South China Seas
9	*Chelon haematocheilus*	*D-Loop*	Yes	3	Pleistocene isolation in the Northwestern Pacific marginal seas and limited dispersal in a marine fish, *Chelon haematocheilus* (Temminck & Schlegel, 1845)
10	*Eriocheir sensu stricto*	*AFLP*	Yes	7	Genome scan of the mitten crab *Eriocheir sensu stricto* in East Asia: Population differentiation, hybridization and adaptive speciation
11	*Portunus sanguinolentus*	*D-Loop*	Yes	6	Phylogeography and Genetic Structure of the Swimming Crabs *Portunus sanguinolentus* (Herbst, 1783) in East Asia
12	*Scylla paramamosain*	*COI+16S rDNA*	Yes	10	Late Pleistocene population expansion of *Scylla paramamosain* along the coast of China: A population dynamic response to the Last Interglacial sea level highstand
13	*Liza affinis*	*SSR*	Yes	8	Population Genetic Structure of *Liza affinis* (Eastern Keelback Mullet), Reveals High Gene Flow Inferred from Microsatellite Analysis
14	*Periophthalmus modestus*	*ND5*	No	5	Biogeographical role of the Kuroshio Current in the amphibious mudskipper *Periophthalmus modestus* indicated by mitochondrial DNA data
15	*Platypleura hilpa*	*COI+COII+ Cytb*	Yes	24	Phylogeography of the cicada *Platypleura hilpa* in subtropical and tropical East Asia based on mitochondrial and nuclear genes and microsatellite markers
16	*Atrina pectinata*	*COI*	No	10	Population genetic structure of the pen shell *Atrina pectinata* sensu lato (Bivalvia: Pinnidae) throughout East Asia
17	*Siphonaria japonica*	*COI*	No	5	Causations of phylogeographic barrier of some rocky shore species along the Chinese coastline
18	*Siphonaria japonica*	*ITS*	No	5	Causations of phylogeographic barrier of some rocky shore species along the Chinese coastline

19	*Macrobrachium nipponense*	*COI+16S rRNA*	Yes	6	Phylogeography and genetic structure of the oriental river prawn *Macrobrachium nipponense* (Crustacea: Decapoda: Palaemonidae) in East Asia
20	*Cyclina sinensis*	*COI*	Yes	9	Phylogeography of Bivalve *Cyclina sinensis*: Testing the Historical Glaciations and Changjiang River Outflow Hypotheses in Northwestern Pacific
21	*Cyclina sinensis*	*ITS-1*	Yes	9	Phylogeography of Bivalve *Cyclina sinensis*: Testing the Historical Glaciations and Changjiang River Outflow Hypotheses in Northwestern Pacific
22	*Tachypleus tridentatus*	*AT-rich region*	Yes	8	Phylogeography, Demographic History, and Reserves Network of Horseshoe Crab, *Tachypleus tridentatus*, in the South and East China Seaboards
23	*Penaeus japonicus*	*COI*	Yes	5	Molecular population structure of the kuruma shrimp *Penaeus japonicus* species complex in western Pacific
24	*Penaeus japonicus*	*D-Loop*	Yes	5	Molecular population structure of the kuruma shrimp *Penaeus japonicus* species complex in western Pacific
25	*Penaeus japonicus*	*SSR*	No	5	Molecular population structure of the kuruma shrimp *Penaeus japonicus* species complex in western Pacific
26	*Eleutheronema rhadinum*	*COI*	Yes	4	Population genetic characteristics of *Eleutheronema rhadinum* and *Eleutheronema tetradactylus* in China (in Chinese)
27	*Eleutheronema rhadinum*	*D-Loop*	Yes	4	Population genetic characteristics of *Eleutheronema rhadinum* and *Eleutheronema tetradactylus* in China (in Chinese)
28	*Arcotheres sinensis*	*COI*	Yes	4	Morphological variation and population genetic structure of the crab *Arcotheres sinensis* from coastal China (in Chinese)
29	*Parasesarma affinis*	*COI*	No	12	Studies on molecular phylogeny of sesarmid crabs from the coast of China and molecular phylogeogrphy of two mangrove crabs (in Chinese)
30	*Octopus ovulum*	*Cyt b*	No	4	Analysis of genetic diversity and structure of octopuses (in Chinese)
31	*Sthenoteuthis oualaniensis*	*Cytb*	Yes	2	Genetic structure of *Sthenoteuthis oualaniensis* population in the northwest Pacific ocean (in Chinese)
32	*Pagrus major*	*D-Loop*	Yes	9	Genetic diversity of 3 sparid species in coastal waters of China based on

					mitochondrial control region sequences (in Chinese)
33	*Acanthopagrus schlegeli*	*D-Loop*	Yes	11	Genetic diversity of 3 sparid species in coastal waters of China based on mitochondrial control region sequences (in Chinese)
34	*Rhabdosargus sarba*	*D-Loop*	Yes	6	Genetic diversity of 3 sparid species in coastal waters of China based on mitochondrial control region sequences (in Chinese)
35	*lisha elongate*	*D-Loop*	Yes	8	Population genetics of the *Ilisha elongata* (Clupeiformes: Pristigateridae) along the Northwestern Pacific coast and the development of SNP marker for *Ilisha elongata* and coilia nusus (in Chinese)
36	*Oratosquilla oratoria*	*ND5+COI+COB*	Yes	6	Phylogeography of Manatosquilla shrimp populations and molecular phylogeny of Sternoporus in the coastal mouth of China (in Chinese)
37	*Pampus chinensis*	*D-Loop*	No	2	Studies on Morphology and genetics of Pampus species (in Chinese)
38	*Pampus minor*	*D-Loop*	Yes	2	Studies on Morphology and genetics of Pampus species (in Chinese)
39	*Cyclina sinensis*	*COI*	Yes	9	Phylogeography of four marine bivalves along China' coastline, with views into the evolutionary processes and mechanisms (in Chinese)
40	*Tegillarca granosa*	*COI*	Yes	9	Phylogeography of four marine bivalves along China' coastline, with views into the evolutionary processes and mechanisms (in Chinese)
41	*Scomber japonicus*	*D-Loop*	No	5	Molecular phylogeography of two scomber species in Northwestern Pacific (in Chinese)
42	*Scomber japonicus*	*SSR*	Yes	5	Molecular phylogeography of two scomber species in Northwestern Pacific (in Chinese)
43	*Konosirus punctatus*	*D-Loop+COI*	Yes	3	Population genetic structure and molecular phylogeography of *Konosirus punctatus* and Sardinella zunasi (in Chinese)
44	*Konosirus punctatus*	*AFLP*	Yes	3	Causations of phylogeographic barrier of some rocky shore species along the Chinese coastline
45	*Konosirus punctatus*	*ISSR*	Yes	2	Causations of phylogeographic barrier of some rocky shore species along the Chinese coastline

Table S2. The information of carapace width, carapace length, abdomen width, abdomen length, weight and gender.

Population	Carapace width (cm)	Carapace length (cm)	Abdomen width (cm)	Abdomen length (cm)	Weight (g)	Female: Male
MA	2.04 ± 0.36	1.71 ± 0.34	0.67 ± 0.24	1.41 ± 0.32	5.41 ± 2.25	5:10
CW	3.09 ± 0.34	2.64 ± 0.23	1.44 ± 0.47	2.31 ± 0.26	16.59 ± 5.40	17:7
DT	3.23 ± 0.35	2.72 ± 0.37	1.38 ± 0.55	2.44 ± 0.37	18.06 ± 6.17	14:6
DZG	1.99 ± 0.29	1.72 ± 0.27	0.65 ± 0.21	1.40 ± 0.29	5.28 ± 2.51	7:14
MZD	2.94 ± 0.20	2.47 ± 0.21	1.16 ± 0.38	2.18 ± 0.19	13.38 ± 2.66	9:7
QA	2.94 ± 0.26	2.44 ± 0.24	1.22 ± 0.49	2.11 ± 0.23	15.01 ± 3.54	15:7
NJLD	3.11 ± 0.40	2.65 ± 0.36	1.42 ± 0.49	2.39 ± 0.47	16.15 ± 6.95	8:2
PT	2.95 ± 0.35	2.45 ± 0.30	1.19 ± 0.51	2.14 ± 0.31	13.68 ± 4.54	10:8
ZJS	3.40 ± 0.29	2.86 ± 0.35	1.48 ± 0.51	2.57 ± 0.34	22.82 ± 7.11	9:5
DH	3.07 ± 0.27	2.49 ± 0.25	1.10 ± 0.61	2.27 ± 0.30	14.43 ± 4.56	7:8
SYH	2.21 ± 0.32	1.93 ± 0.26	0.81 ± 0.36	1.67 ± 0.31	5.54 ± 2.50	5:10
XM	3.06 ± 0.32	2.58 ± 0.28	1.31 ± 0.47	2.26 ± 0.33	16.65 ± 5.72	13:5
FYLD					16.07 ± 3.32	7:8
HA					13.42 ± 2.33	8:7
JH					14.03 ± 2.41	2:3

Table S3. Pairwise F_{ST} values between populations based on *COI* sequences. All pairwise F_{ST} values between populations were not statistically significant ($p > 0.05$).

	JH	PT	ZJS	SYH	MA	HA	XM	CW	DT	FYLD	DH	DZG	MZD	QA	NJLD
JH	0.0000														
PT	0.0054	0.0000													
ZJS	−0.0370	−0.0328	0.0000												
SYH	−0.0134	0.0025	−0.0124	0.0000											
MA	−0.0147	0.0070	−0.0273	0.0177	0.0000										
HA	−0.0412	0.0068	−0.0308	−0.0116	−0.0129	0.0000									
XM	0.0358	0.0138	0.0162	0.0602	−0.0003	0.0321	0.0000								
CW	−0.0039	0.0040	−0.0249	0.0433	−0.0315	−0.0018	0.0107	0.0000							
DT	−0.0232	0.0094	−0.0290	0.0170	−0.0327	−0.0054	0.0087	−0.0133	0.0000						
FYLD	−0.0131	0.0076	−0.0170	0.0329	−0.0163	0.0124	0.0229	−0.0123	−0.0091	0.0000					
DH	0.0065	0.0115	−0.0168	0.0234	−0.0402	0.0063	−0.0213	−0.0149	−0.0269	0.0066	0.0000				
DZG	−0.0190	−0.0106	−0.0353	0.0105	−0.0076	−0.0047	0.0507	−0.0134	0.0007	−0.0247	0.0239	0.0000			
MZD	−0.0275	0.0062	−0.0212	0.0109	0.0065	−0.0182	0.0526	0.0025	0.0038	−0.0188	0.0345	−0.0201	0.0000		
QA	−0.0240	−0.0034	−0.0400	0.0119	−0.0230	−0.0146	0.0261	−0.0192	−0.0310	−0.0066	−0.0131	−0.0143	−0.0122	0.0000	
NJLD	−0.0116	−0.0456	−0.0406	−0.0130	0.0033	−0.0087	0.0181	−0.0013	0.0041	−0.0245	0.0152	−0.0393	−0.0359	−0.0111	0.0000

Table S4. Pairwise F_{ST} values between populations based on *D-Loop* sequences. The genetic differentiation between the remaining populations showed insignificant differences ($p > 0.05$), except for the significant differences between MZD and FYLD, and between MZD and HA populations ($p < 0.05$).

	MA	DT	FYLD	MZD	NJLD	PT	ZJS	HA	XM	CW	QA	DH	JH	DZG	SYH
MA	0.0000														
DT	−0.0417	0.0000													
FYLD	−0.0651	0.0139	0.0000												
MZD	0.0519	0.0704	0.1889	0.0000											
NJLD	−0.0482	−0.0933	0.0172	0.0110	0.0000										
PT	−0.0359	−0.0330	0.0906	−0.0284	−0.0688	0.0000									
ZJS	−0.0547	−0.0290	0.0259	−0.0155	0.0625	−0.0260	0.0000								
HA	−0.0436	0.0488	−0.0716	0.2675	0.0552	0.1571	0.0640	0.0000							
XM	−0.0214	−0.0298	0.0490	−0.0477	−0.0699	−0.0451	−0.0571	0.1024	0.0000						
CW	−0.0692	−0.0684	−0.0479	0.1071	−0.0672	0.0219	−0.0161	−0.0460	−0.0023	0.0000					
QA	−0.0325	−0.0591	0.0200	0.1287	−0.0213	0.0129	−0.0107	0.0286	0.0295	−0.0397	0.0000				
DH	−0.0974	0.0121	−0.0628	0.0725	−0.0135	0.0386	−0.0619	−0.0286	−0.0013	−0.0386	−0.0489	0.0000			
JH	−0.0838	−0.0327	0.0351	−0.0541	−0.0918	−0.0910	−0.0514	0.0603	−0.0425	−0.0276	−0.0102	−0.0480	0.0000		
DZG	−0.0988	−0.0797	−0.0705	0.1537	−0.0397	0.0451	−0.0386	−0.0709	−0.0012	−0.1128	−0.0739	−0.0602	−0.0143	0.0000	
SYH	−0.0247	−0.0422	0.1111	−0.0545	−0.0350	−0.0737	−0.0524	0.1410	−0.0512	0.0292	0.0384	0.0330	−0.0536	0.0198	0.0000

Table S5. The effective population sizes for each population based on *COI* and *D-Loop* sequences.

Population	*COI*	*D-Loop*
MA	82784000	127069000
ZJS	84495000	79514000
HA	40708000	60300000
DT	90766000	103192000
NJLD	41633000	106242000
FYLD	2477000	108329000
PT	88047000	119829000
MZD	2060000	15060000
CW	100252000	142173000
XM	11573000	102886000
QA	115895000	132202000
DH	80819000	130186000
JH	39004000	115484000
DZG	34860000	98923000
SYH	40811000	4266000

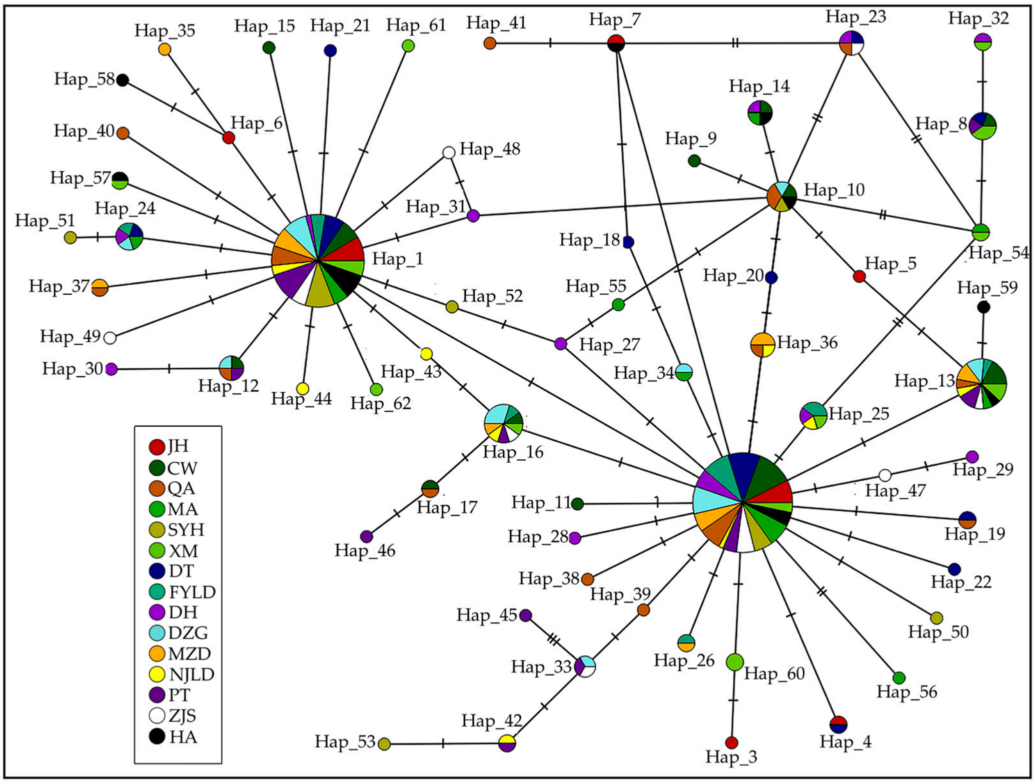

Figure S1. The minimum spanning network based on *COI* sequences.

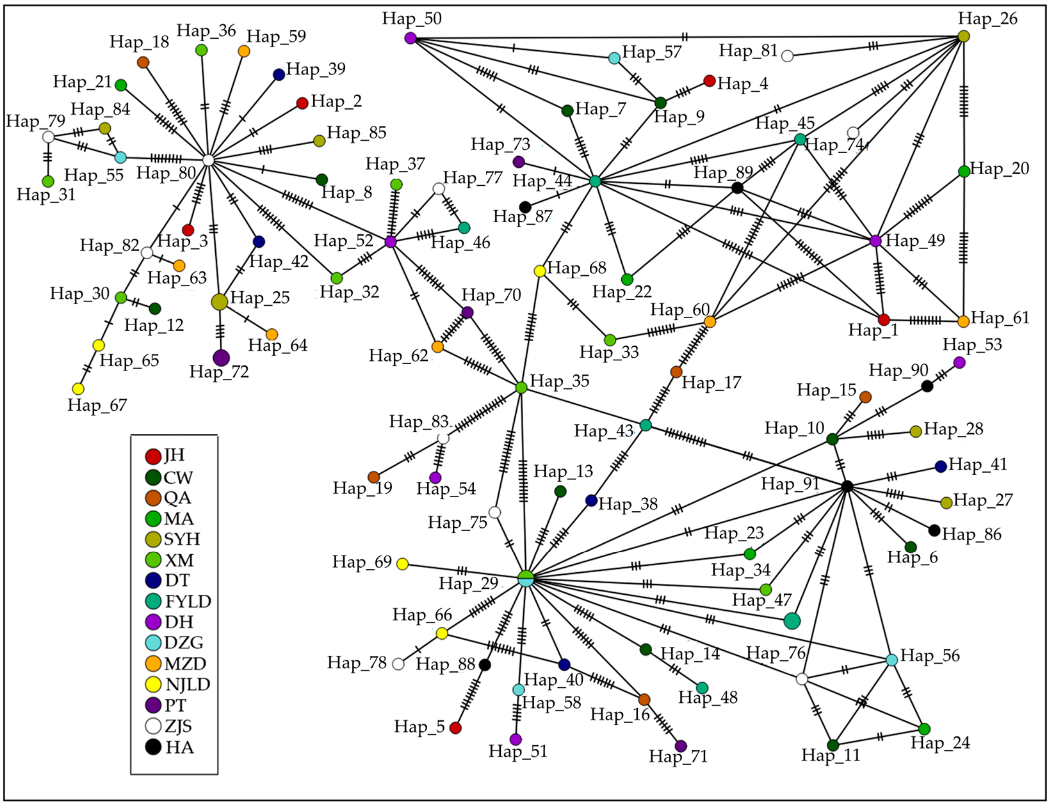

Figure S2. The minimum spanning network based on *D-Loop* sequences.
